# Intelligent Terrestrial and Non-Terrestrial Vehicular Networks with Green AI and Red AI Perspectives

**DOI:** 10.3390/s23020806

**Published:** 2023-01-10

**Authors:** Hyunbum Kim, Jalel Ben-Othman, Lynda Mokdad

**Affiliations:** 1 Department of Embedded Systems Engineering, Incheon National University, Incheon 22012, Republic of Korea; 2 Laboratoire des Signaux et Systémes, CNRS, CentraleSupélec, Université Paris-Saclay, 91190 Gif-sur-Yvette, France; 3 LACL Laboratory, Department of Computer Science, University of Paris-Est, 94000 Créteil, France

**Keywords:** 6G, virtual emotion, UAVs, Red AI, Green AI

## Abstract

In this paper, we aim to envision 6G convergent terrestrial and non-terrestrial infrastructure of virtual emotion and epidemic prevention with two differential perspectives: Green AI and Red AI, where Green AI focuses on efficiency and reduction, and Red AI additionally pursues accuracy. By fitting with each perspective, we introduce promising key applications using smart devices, autonomous UAVs, mobile robots and subsequently suggest critical future research directions and opportunities toward new frontiers in intelligent terrestrial and non-terrestrial vehicular networks.

## 1. Introduction

Fifth generation (5G) wireless and mobile systems have attracted long-standing interest from researchers owing to the clear advantages of 5G new radio milestone, realization of various high-frequency services, and security [1,2,3]. However, because of the massive data-intensive applications including augmented reality, brain interface, and remote surgery in future smart cities, the latency and data rate requirements of those promising applications should be beyond that of current 5G technologies. To overcome the inherent restrictions and drawbacks of 5G, the 6G (sixth generation) system is emerging as a key enabler with higher rates, massive antennas, expanded AI operations, routing algorithms, distributed structures, integrated terrestrial, and airborne features [4,5,6,7,8]. Specifically, in relation to 5G and 6G technologies, the terrestrial, non-terrestrial, space–air–terrestrial networks are attracting much interest from researchers [9,10].

The remarkable concept of *virtual emotion* was originally proposed by [11]. Through smart devices equipped with wireless transmitters and receivers, the virtual emotion is detected by wireless signals and their reflections, and derivation procedures including heartbeat segmentation and respiration procedure by [12]. Then, the *virtual emotion* can be sent to other entities in the form of manipulatable data throughout AI-supported systems. Thanks to its promising applicabilities with various emotion-based services in smart cities, *virtual emotion* is attracting much interest and is continuously expanding its applicable research areas including 5G, security, UAVs (Unmanned Aerial Vehicles), IoT (Internet of Things) devices, epidemic prevention, etc. [13,14].

On the other hand, it is vital to develop these advanced systems for epidemic prevention in order to protect the wellbeing of citizens. Furthermore, it is verified that prompt epidemic prevention is indispensable to restrict unprecedented epidemic spread. Subsequently, we can consider that the utilization of autonomous UAVs, mobile robots and smart devices based on AI-supported 6G communications will play a role in preventing potential disease outbreak and spread because AI-enabled 6G infrastructure will motivate a massive amount of detected data and manipulated information by smart devices to be rapidly communicated as well as perform appropriately [15,16,17,18,19,20,21,22]. Based on the motivation for epidemic prevention, there arises the issue of how to utilize the massive number of heterogeneous system components in a careful manner.

**Figure 1:**
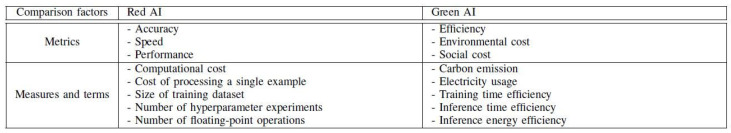
Comparison of Red AI and Green AI.

Moreover, it is highly anticipated that the AI technologies including deep learning and machine learning will be driven by a wide range of innovative applications and systems such as 5G, edge computing, cloud, Internet of Things (IoT), tactile internet, wireless networks, vehicles, blockchain, etc. [23,24,25,26,27,28,29,30,31,32,33,34]. Furthermore, several standpoints are studied regarding AI technology and its applicability. In particular, the *Red AI* and the *Green AI* were defined by [35] recently. The *Red AI* comprises existing AI techniques and approaches which covers accuracy improvement, process speed, and minimum delay to perform specific missions and system goals as main metrics or factors. On the contrary, with another AI-enabled approach, *Green AI* focuses on the economic, environmental and social costs of satisfying system requirements or task completions as evaluation metrics or standards. Figure 1 shows a brief comparison between *Red AI* and *Green AI* with their metrics, measures and used terms. In short, *Green AI* focuses on efficiency and reduction in cost while *Red AI* pursues improvement of accuracy. Subsequently, toward balanced realization for AI-enabled applications, the issue of how to conduct AI-assisted virtual emotion and epidemic prevention infrastructures consisting of heterogeneous components has to be critically addressed.

Based on the above observation, the main contributions of the paper are summarized as follows.

- We design 6G convergent terrestrial and non-terrestrial infrastructure with two differential perspectives: *Red AI* and *Green AI* to manage virtual emotion and epidemic prevention. Furthermore, the considered terrestrial and non-terrestrial regions connect a broad view between space–air–terrestrial network and space–air–non-terrestrial network.
- With *Red AI* perspective, the sub-system 1 is introduced with the below contributions.
  – For the purpose of virtual emotion and epidemic prevention, the promising 6G applications with essential system requirements and operations are specified clearly.
  – The proposed sub-system deliberates on the applicability of 6G technology with higher rates, expanded AI operations, improved synchronization between airborne and ground communication infrastructure with Red AI view.
  – Open research challenges and issues are addressed within *Red AI*-based framework.
- With *Green AI* standpoint, the sub-system 2 is proposed with the following contributions.
  – We shape *Green AI* features and properties regarding virtual emotion and epidemic prevention with greener and broader views. Furthermore, with regard to virtual emotion and epidemic prevention, the new category of 6G services including system settings and operation scenarios is explained.
  – Furthermore, the devised sub-system covers the advantages of 6G technology with Green AI features.
  – We then provide research roadmaps, influential open research issues, and recommendations which can be studied from a *Green AI* perspective.

**Figure 2:**
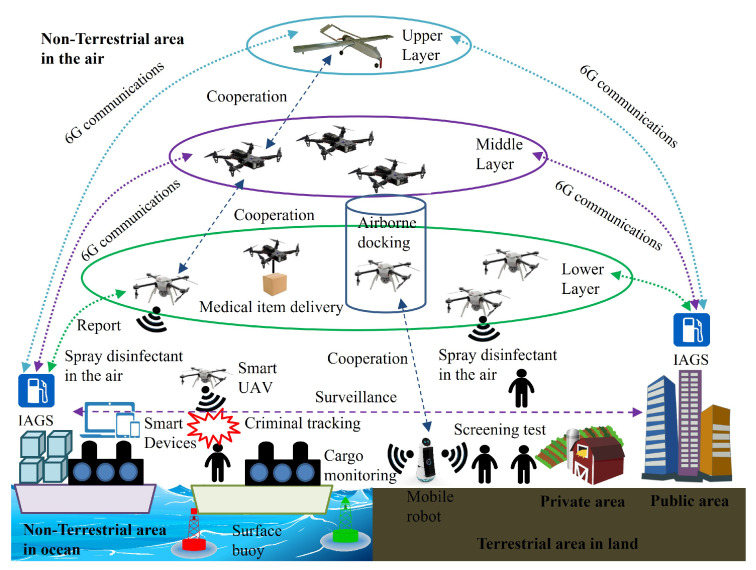
An example of system components and their activities in 6G-supported terrestrial and non-terrestrial areas.

**Figure 3:**
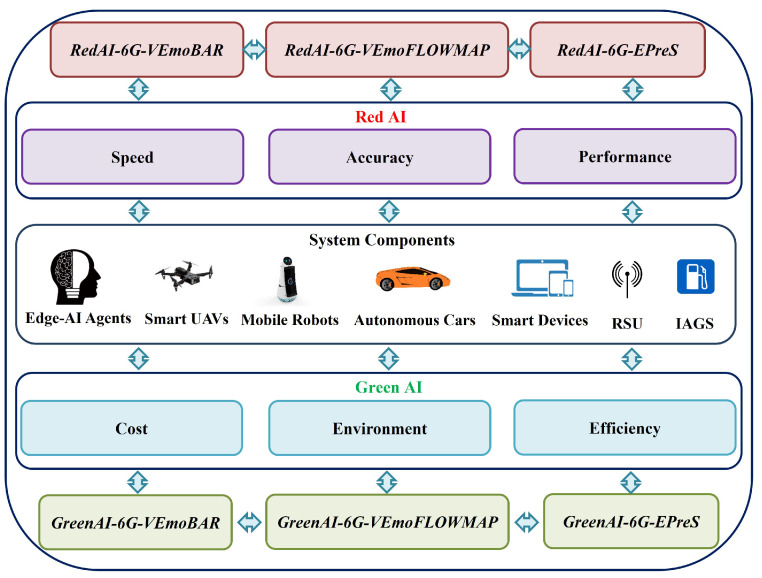
A brief overview of the proposed 6G convergent infrastructure with two differential perspectives: *Red AI* and *Green AI*.

Figure 2 depicts system components, activities in terrestrial and non-terrestrial areas which cover aerial side, extended ocean, and land with public and private areas. Furthermore, Figure 3 presents a brief overview of the designed 6G convergent infrastructure with two different views of *Red AI* and *Green AI*, covering the proposed two sub-systems, their components, and critical features of *Red AI* and *Green AI*.

## 2. 6G Convergence with Red AI Perspective in Terrestrial and Non-Terrestrial Applications

In this section, by borrowing from the concepts of *virtual emotion barrier*, *virtual emotion flow*, and *virtual emotion map* [11,13], we introduce *Red-AI-enabled virtual emotion barrier* and *Red-AI-enabled 6G virtual emotion flow and map* with a view of *Red AI* that focuses on improved accuracy, process speed, minimum delay rather than economic and environmental costs. Furthermore, *Red-AI-enabled 6G epidemic prevention service* is described to utilize mobile robots and autonomous UAVs in a 6G environment.

### 2.1. Application 1: Red-AI-Enabled 6G Virtual Emotion Barrier

*Definition*: *Red-AI-Enabled 6G Virtual Emotion Barrier*, referred to as *RedAI-6G-VEmoBAR*, is a barrier with a *Red AI* view that is able to detect human emotion types such as joy, pleasure, neutrality, sadness, fury and concentrates on accuracy and speed in a 6G environment. Given that a set of heterogeneous devices including UAVs, mobile robots, smart devices equipped with wireless transmitters and reflection receivers in a 6G-supported square-shaped area, *RedAI-6G-VEmoBAR* detects the specific emotion type of any person passing through a given field.

*System Goal*: As an ultimate system goal and maintenance, *RedAI-6G-VEmoBAR* aims to increase system performance and then eventually provide virtual-emotion-based AI services to residents in smart cities. The first objective is to maximize system lifetime by building multiple *RedAI-6G-VEmoBAR* instead of single barrier construction. The second aim is maximizing emotion detection accuracy. Furthermore, the third objective is to concentrate on minimizing recovery delay from failure of barriers regardless of energy consumption of devices.

*Operation*: According to [12], the system assumes that human emotions such as joy, pleasure, neutral, sadness, and fury can be detected through wireless signals, their reflections, camera sensor, temperature sensor, heartbeat segmentation, derivation procedures with heartbeat segmentation and respiration signals. Furthermore, we can consider that several group of devices including a fleet of UAVs, and a group of mobile robots, smart devices have random locations initially where UAVs are randomly deployed in the air as well as mobile robots and smart devices that are active on the ground. According to service type and purpose, the formed barriers detect the heartbeat segmentation, respiration signal and body temperature through wireless signal and its reflection, and sensors. It follows that the virtual emotion information and the feature for epidemic prevention are derived. That is, the specific derived information can be focused differently. For example, if the surveillance and patrol service based on virtual emotion information are required for specific region, the proposed barrier system focuses on the detection of fury emotion type in order to prevent potential criminal and terror threat. Furthermore, an *Integrated Aerial–Ground Station*, referred to as *IAGS*, is installed in 6G convergent infrastructure to recharge UAVs, mobile robots, autonomous vehicles and smart devices during staying *IAGS*. Because sleep–wake scheduling can be applied alternately among multiple barriers, the multiple numbers of *RedAI-6G-VEmoBAR* are formed by heterogeneous devices to maximize system life. Then, the detected virtual emotion information through *RedAI-6G-VEmoBAR* is stored. Subsequently, the large amount of accumulated data is sent to other entities near devices or the closest edge-AI agent with a large amount of data implementing deep learning so that AI-assisted virtual emotion services can be provided properly. It is noted that the virtual emotion barrier is composed of several system devices using wireless signal, and the accuracy of detection through wireless signal and its reflection depends on the distance between device and person. So, as one of the metrics for accuracy in virtual emotion barrier, the accumulated accuracy estimated by each pair between each device in the barrier can be considered. Hence, the barrier with high accumulated accuracy by devices is chosen from multiple barriers with priority so that the system increases the accumulative accuracy as a whole in the given area with a view of *Red AI*. Furthermore, after constructing multiple *RedAI-6G-VEmoBAR*, the mobile system components of smart devices, UAVs, mobile robots may move to other locations or may have failures so that those failures will cause weak detection points and holes in *RedAI-6G-VEmoBAR*. To resolve the issue, a fleet of UAVs with fast moving speed are utilized to fill the detection hole in the barrier where the system requires rapid recovery from the failures. Otherwise, mobile robots with more powerful resources and larger detection ranges can be used to recover the weak points of *RedAI-6G-VEmoBAR*.

### 2.2. Application 2: Red-AI-Enabled 6G Virtual Emotion Flow and Map

*Definition*: *Red-AI-Enabled 6G Virtual Emotion Flow and Map*, referred to as *RedAI-6G-VEmoFLOWMAP*, is a map with flow by *Red AI* view that can be derived from the accumulated and the detected virtual emotion information by already formed *RedAI-6G-VEmoBAR* with 6G communications. *RedAI-6G-VEmoFLOWMAP* is generated for specific emotion type during specific period and at specific region. Furthermore, the requested detection accuracy and the given request response speed are considered as system performance.

*System Goal*: Ultimately, *RedAI-6G-VEmoFLOWMAP* aims to increase the performance of the system such as delay, creation accuracy, proper virtual emotion service matching. So, the first objective is to minimize derivation and creation delay of *RedAI-6G-VEmoFLOWMAP*. The second purpose is to maximize the creation accuracy of *RedAI-6G-VEmoFLOWMAP* for specific emotion type. The third goal is to maximize virtual emotion service matching correctness to people appropriately with anonymity and security for people.

*Operation*: From *RedAI-6G-VEmoBAR*, AI agents at edge computing level receive accumulated virtual emotion data sets. It follows that we collect a large amount of virtual emotion information through autonomous vehicles, UAVs and smart devices in public roads, building, institutions, then the flow and map of specific emotion type is created so that the independent *RedAI-6G-VEmoFLOWMAP* for each emotion type can be generated as well as the complex *RedAI-6G-VEmoFLOWMAP* representing several emotion types can be obtained. With *Red AI* view, the *RedAI-6G-VEmoFLOWMAP* aims to increase the accuracy of virtual emotion derivation by deliberating on extra computations and expanded amount of information. For operations, there are available service cases based on virtual emotion such as current emotion flow check, criminal prevention, virtual-emotion-based advertisement, violent driver tracking, etc. For example, some pedestrians in public streets make requests for *RedAI-6G-VEmoFLOWMAP* to the system to monitor current status of neighbor places whether there are potential criminals due to unstable emotion types such as fury and rage, the closest AI agents then send the *RedAI-6G-VEmoFLOWMAP* to those pedestrians in response. If a sole AI agent bears a large burden from numerous requests through 6G convergent system, the request can be forwarded to next closest AI agent gradually. Because the reliability of system depends on the derived accuracy, *RedAI-6G-VEmoFLOWMAP* focuses on how to increase the accuracy. Since the virtual emotion information of people is changed frequently, the large amount of virtual emotion data should be collected and treated repeatedly.

### 2.3. Application 3: Red-AI-Enabled 6G Epidemic Prevention Service

*Definition*: *Red-AI-Enabled 6G Epidemic Prevention Service*, marked as *RedAI-6G-EPreS*, is a service based on 6G communications and *Red AI* view that centralizes upon accuracy, speed and provides epidemic services covering rapid medical item delivery, epidemic prevention visit, epidemic prevention map creation, etc. The *RedAI-6G-EPreS* is supported by aerial–ground cooperation including UAVs in the air, mobile robots, smart devices, and autonomous vehicles on the ground.

*System Goal*: The ultimate goal of *RedAI-6G-EPreS* is to support epidemic prevention and to suppress pandemic spread Firstly, *RedAI-6G-EPreS* deliberates on minimizing medical item delivery delay of UAVs and mobile robots from current locations to requested delivery service regions without any collisions among multiple UAVs and mobile robots. Secondly, *RedAI-6G-EPreS* focuses on how to improve the completion time of epidemic prevention service and the prevention service. Thirdly, *RedAI-6G-EPreS* makes good progress of epidemic service accuracy by system or users so that the scheduled various services should be completed correctly to satisfy specific accurate rate for requested tasks.

*Operation*: Initially, the monitoring region of interests and the requested service area are identified by reported information from numerous smart devices, users, UAVs, mobile robots, autonomous devices with 6G-assisted communications during specific time. The *RedAI-6G-EPreS* is performed at two different types: public area and private area. The public area allows the access for all kinds of devices. However, private area requires the access permission and then, only devices with permission can enter into private locations. With 6G communications and deep learning algorithms, AI agents decide which UAVs fly to the requested service area to deliver light-weighted medical materials such as masks, sterilizer, sanitary cup and also determine which mobile robots move to service locations to deliver relatively heavy-weighted epidemic prevention items such as protective gear, disinfected overgarment, etc. Then, AI agents not only create the matching schedule between available UAVs, mobile robots and requested services but also find the minimum distance trajectory for the mission with minimum delivery delay because the requested service can be emergent at harsh environment. In order to suppress pandemic outbreak, the epidemic prevention visit is mandatory and it should fulfill the standard of non-face-to-face or non-contact. So, after getting the permission into private fields and homes, the cooperation between mobile robots and UAVs in 6G convergent environment will be helpful for those epidemic visit missions of quarantine, visit screening tests, non-contact prescriptions of medical doctors which are non-face-to-face medical services such as mobile health application, platform. On the other hand, both UAVs and mobile robots can be utilized to spray disinfectant into target public and private areas rapidly. Hence, the cooperation among UAVs, mobile robots and other smart devices with 6G communications will contribute to several goals such as minimizing delivery delay, reducing epidemic prevention completion time, maximizing the throughput of required epidemic mission tasks.

**Figure 4:**
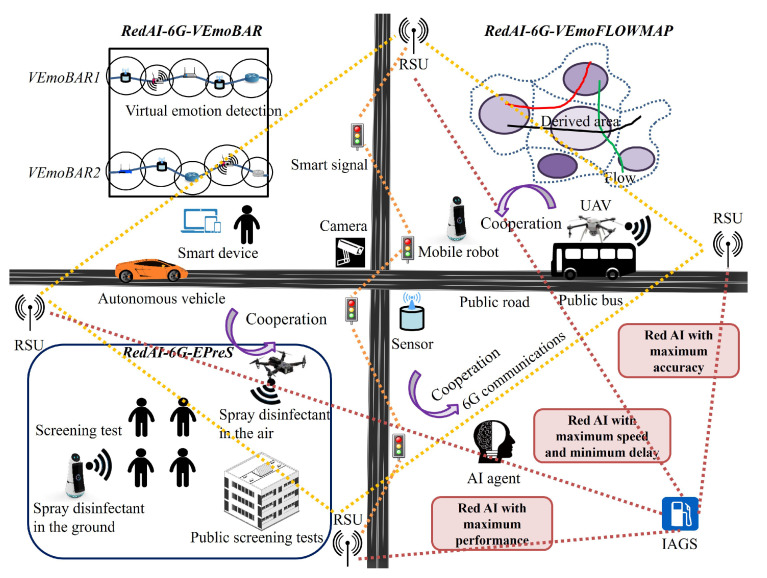
A depiction of applications with main components, operations and objectives including *RedAI-6G-VEmoBAR*, *RedAI-6G-VEmoFLOWMAP*, and *RedAI-6G-EPreS* with *Red AI* view.

Figure 4 depicts the proposed applications of *RedAI-6G-VEmoBAR*, *RedAI-6G-VEmoFLOWMAP*, and *RedAI-6G-EPreS Red AI* view. It pursues to accomplish maximum accuracy, maximum speed, minimum delay, maximum performance for those applications.

## 3. Open Research Issues toward 6G Convergent Red AI

### 3.1. Open Research Issue 1 of 6G Convergent Red AI

Firstly, the proposed 6G convergent system with *Red AI* view is dependent on the accuracy of detection and creation for *RedAI-6G-VEmoBAR*, *RedAI-6G-VEmoFLOWMAP*, respectively. The detection accuracy depends on the distance between wireless devices and target person. So, because the detection accuracy by the wireless signal and its reflection is dropped as the distance between devices and person is diminished at the detection moment, the practical approaches and its implementation standards to return high detection accuracy of virtual emotion should be studied continuously. Furthermore, various tasks at *RedAI-6G-EPreS* can be progressed by UAVs and mobile robots. In particular, a fleet of UAVs can be utilized to deliver medical items and to spray disinfectant into target regions. During mission achievement, there is a potential possibility of collisions among multiple group of components. Those collisions will bring serious problems such as failures, performance degradation by faults and people may be wounded due to near collisions among UAVs, mobile robots in the aerial-ground sides. As initial work of 6G convergent, it is also necessary to consider how to synchronize other relevant technologies such as autonomous vehicles and robots. So, we need to make standard rules of liability in case of those collision situations and then, we should develop practical schemes which can resolve collision problems completely for stable realization of the 6G convergent system supported by heterogeneous aerial and ground devices.

### 3.2. Open Research Issue 2 of 6G Convergent Red AI

Secondly, we point out the security issue as critical open research issue for secure implementation of 6G convergent system consisting of *RedAI-6G-VEmoBAR*, *RedAI-6G-VEmoFLOWMAP*, *RedAI-6G-EPreS*. Since the emotion detection is implemented through wireless signal at *RedAI-6G-VEmoBAR*, the malicious attackers may forge wireless signal or perform illegal disruption of signal by hijacking. Those illegal forgeries and modifications may have serious security threats and lead the system performance degradation. Then, if the illegally modified signals cause wrong derivation outcomes, they will affect incorrect creation of *RedAI-6G-VEmoFLOWMAP* so that the reliability of the whole system can be ruined due to numerous fake emotion-based services. Furthermore, for *RedAI-6G-EPreS*, the tampered signals may influence the system performance diminishment through wrong information transmission and its immediate dissemination in 6G environment. Furthermore, the compromised devices including autonomous vehicles, mobile robots, UAVs will give unexpected malfunctions or unknown misbehaviors. On the other hand, the proposed system requires the anonymity for detected virtual emotion information of citizens and the secure access control for publica area and private area. Therefore, secure AI-based deep learning and machine learning solutions and privacy-preserving access controls should be devised to preserve 6G convergent system from forgery, hijacking, zero-day attack, malfunctions by compromised components.

## 4. 6G Convergence with Green AI Perspective in Terrestrial and Non-Terrestrial Applications

In this section, with alternative view of *Green AI*, we envision *Green-AI-Enabled 6G Virtual Emotion Barrier*, *Green-AI-enabled 6G virtual emotion flow and map*, *Green-AI-enabled 6G epidemic prevention service*, which seek task accomplishment, efficiency of computational expense, environment protection, reduction in spent resources.

### 4.1. Application 1: Green AI Enabled 6G Virtual Emotion Barrier

*Definition*: *Green-AI-Enabled 6G Virtual Emotion Barrier*, referred as *GreenAI-6G-VEmoBAR*, is a barrier with a *Green AI* view, which specific emotion type is detected by a series of heterogeneous devices consisting of UAVs, mobile robots, smart devices. Furthermore, it focuses on economic, environmental, social cost in 6G environment.

*System Goal*: Basically, *GreenAI-6G-VEmoBAR* looks for favorable trade-off between performance and efficiency by identifying key factors of task accomplishment, computational cost with minimum reduction in performance, environment protection, reduction in spent resources. The first objective is to minimize computation cost for the number of devices in *GreenAI-6G-VEmoBAR*, which meet the given emotion detection accuracy requirement toward appropriate emotion based services in smart cities. The second goal is to reduce 6G communication resources such as communication messages, transmitted signals when *GreenAI-6G-VEmoBAR* is activated in the given area. Furthermore, the third aim is to minimize energy expenditure when *GreenAI-6G-VEmoBAR* is constructed as well as is restored from failures.

*Operation*: The system assumes that the basic human emotion types for joy, pleasure, neutral, sadness, fury are detected by wireless signal and its reflection. It is given that the heterogeneous components consisting of UAVs, mobile robots, smart devices, AI agent at edge computing in 6G-assisted area have random locations initially. Firstly, the system accepts the required detection accuracy from system administrator or users. Furthermore, in order to build *GreenAI-6G-VEmoBAR*, we take into account the minimum number of elements to schedule which components move from initial positions to specific location within the planned multiple formations of *GreenAI-6G-VEmoBAR* where sleep-wakeup scheduling is applicable on condition that the required detection accuracy is attained. After a multitude of *GreenAI-6G-VEmoBAR* are set up in 6G environment, the detected emotion information by *GreenAI-6G-VEmoBAR* is transmitted to the closest AI agents with reduced 6G communication in spent resources. Then, by the cooperation of multiple AI agents and the implementations of deep/machine learning schemes at edge side, the meaningful emotion information is generated for emotion-based services. Furthermore, due to dynamic movement property of heterogeneous devices in the system, the formed *GreenAI-6G-VEmoBAR* during specific time can be destroyed temporarily. To solve those provisional failures, *GreenAI-6G-VEmoBAR* takes care of minimizing energy consumptions for movements of UAVs, mobile robots and other mobile devices from current locations to failed positions instead of thinking over re-construction delay. It follows that the failure reovery of *GreenAI-6G-VEmoBAR* only deals with energy consumption of heterogeneous devices because the electricity usage of each movable component is different.

### 4.2. Application 2: Green-AI-Enabled 6G Virtual Emotion Flow and Map

*Definition*: *Green-AI-Enabled 6G Virtual Emotion Flow and Map*, denoted as *GreenAI-6G-VEmoFLOWMAP*, is a map with flow by *Green AI* perspective which can be brought from the aggregated and the detected virtual emotion information by the built *GreenAI-6G-VEmoBAR* in 6G environment. For specific emotion type, period and region, *GreenAI-6G-VEmoFLOWMAP* is produced with a consideration of computational efficiency, implementation and operation efficiency as principal factors of the system.

*System Goal*: At *GreenAI-6G-VEmoFLOWMAP*, the first goal is to minimize the total number of floating-point operations, called as *FPO*, which is required when any machine learning algorithm performs abstract operation such as matrix multiplication, convolution operation to derive *GreenAI-6G-VEmoFLOWMAP* from a large amount of emotion detection information by *GreenAI-6G-VEmoBAR*. The second aim is to minimize the total running time of deep and machine learning algorithm so as to generate the adequate *GreenAI-6G-VEmoFLOWMAP*. Furthermore, the third objective is to minimize the number of parameters and required conditions which are claimed to yield *GreenAI-6G-VEmoFLOWMAP* consequently.

*Operation*: Supplied from *GreenAI-6G-VEmoBAR*, AI agents located at edge computing side get the stored virtual emotion information during specific period. It follows that AI agents aggregate a massive amount of virtual emotion data sets from UAVs, mobile robots, autonomous vehicles, smart devices within *GreenAI-6G-VEmoBAR* which are positioned in public areas and private areas with permission. Not only for virtual emotion flow, the flow representing the specific emotion type is appended and but also for virtual emotion map, the region depicting the specific emotion with degree level is added to *GreenAI-6G-VEmoFLOWMAP* essentially. With *Green AI* perspective, AI agents can execute to minimize the total number of floating-point operations and the amount of work done when *GreenAI-6G-VEmoFLOWMAP* is produced. Furthermore, the reduced running time to return *GreenAI-6G-VEmoFLOWMAP* at AI agents should be considered where the runtime can be correlated with *FPO* and the amount of work done while *GreenAI-6G-VEmoFLOWMAP* is created with *Green AI*. Moreover, *GreenAI-6G-VEmoFLOWMAP* is based on many-sided parameters, system settings and status because *GreenAI-6G-VEmoFLOWMAP* have diverse parameters and conditions such as required detection accuracy level, region of interests size and its shape, public/private area, access permission, system budget, system resource limitations, etc.

### 4.3. Application 3: Green-AI-Enabled 6G Epidemic Prevention Service

*Definition*: *Green-AI-Enabled 6G Epidemic Prevention Service*, represented as *GreenAI-6G-EPreS*, is a service supported by 6G infrastructure and *Green AI* perspective, which advocates epidemic prevention service including environmentally friendly medical item delivery, inexpensive epidemic prevention visit, suitable epidemic prevention map creation with the reduced data and the diminished number of training experiments and communications.

*System Goal*: For *GreenAI-6G-EPreS*, the first objective is to reduce environmentally measured factors such as carbon emission by any vehicles and over-sprayed disinfectant, which can be harmful to green circumstances including low carbon emission usage, reduced cost of data processing, reduction in spent resources and environmental hygiene. The second goal is to shorten the cost of hardware and software that can be utilized for system process and implementation for epidemic prevention and disease outbreak suppression. Lastly, the third aim is to economize the massive number of training experiments and underlying simulations for *GreenAI-6G-EPreS*.

*Operation*: At early stage, the environmentally friendly evaluation criterion of carbon emission, air pollution is verified as numerical values and then those values are distributed to all entities including users, AI agents, UAVs, mobile robots, autonomous vehicles and smart devices by 6G-supported communications. Furthermore, those entities identify both public area and private area. While public region is accessed by all kinds of components, private area requires only components with access permission. For medical item delivery of epidemic prevention, AI agents determine which UAVs, vehicles or mobile robots deliver light-weighted items and heavy-weighted item selectively to requested locations to fulfill environmental standard like low carbon emission, saved electricity usage. Basically, epidemic prevention visit covers non-face-to-face visit to specific locations to be served, which are supported by mobile robots on the ground side to deliver a small size of medical items. When the epidemic prevention visit by the cooperation of UAVs and mobile robots is proceeded, the environmental standard should be satisfied for carbon emission by any delivery vehicles, disinfectant sprayed by UAVs in the air. Furthermore, for contributing 6G-assisted epidemic visit tasks such as quarantine, screening test and notifications, non-contact prescriptions, the reduced expense of hardware and software is necessary. In addition, when AI agents calculate the trajectory of delivery and epidemic prevention visit, system recovery from malfunctions, the estimations and outcomes should be gained by economic number of training experiments and reduced relevant information at AI agents, not excessively massive training data sets and information for required measurement.

**Figure 5:**
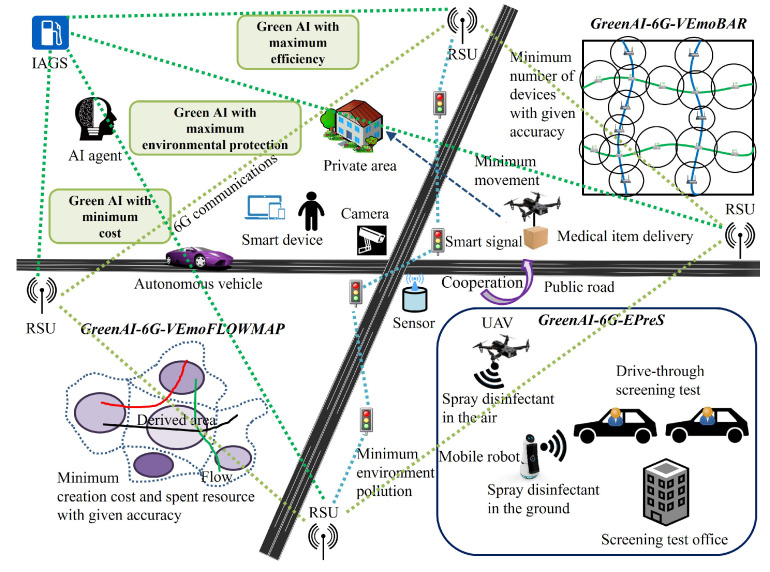
A description of applications with main components, operations and objectives including *GreenAI-6G-VEmoBAR*, *GreenAI-6G-VEmoFLOWMAP*, *GreenAI-6G-EPreS* with *Green AI* view.

Figure 5 shows the proposed applications of *GreenAI-6G-VEmoBAR*, *GreenAI-6G-VEmoFLOWMAP*, *GreenAI-6G-EPreS Green AI* view. It aims to achieve maximum efficiency, maximum environmental protection, and minimum expense for those applications.

## 5. Open Research Issues toward 6G Convergent Green AI

### 5.1. Open research Issue 1 of 6G Convergent Green AI

First, the efficiency should be investigated by researchers to realize *GreenAI-6G-VEmoBAR*, *GreenAI-6G-VEmoFLOWMAP*, *GreenAI-6G-EPreS* ensuring with 6G convergence. In particular, data efficiency can be a primary open research challenge. Although data efficiency has received much attention over the years in other research branches, it is a key consideration when *GreenAI-6G-VEmoBAR*, *GreenAI-6G-VEmoFLOWMAP* and *GreenAI-6G-EPreS* are performed practically through the massive amount of training data including excessive raw and unannotated data. For example, instead of deriving useful emotion information from the large amount of data to fit with virtual emotion services, it is crucial that the meaningful virtual emotion data for possible exact emotion services to citizen should be extracted from a reasonable amount of data and with a reduced amount of training data in experiments. Furthermore, another issue with regard to efficiency is cost efficiency. There are several deep learning/machine learning schemes in existing studies. These existing deep learning/machine learning approaches can be applied or new approaches can be developed to achieve *GreenAI-6G-VEmoBAR*, *GreenAI-6G-VEmoFLOWMAP*, and *GreenAI-6G-EPreS* to cut down the floating-point operations (i.e., matrix multiplication, convolution or tanh operation), the amount of work done and the elapsed running time through various neural libraries, data blocks and applied AI models. Subsequently, how to choose the best adapted deep learning/machine algorithm and AI model should be a significant consideration so as to minimize the expense of the proposed 6G convergent system.

### 5.2. Open Research Issue 2 of 6G Convergent Green AI

Secondly, the research direction of environmentally friendly operation and perfect maintenance is unexplored and has to be studied widely by collaborative researchers and institution teams with diverse researchers such as AI data scientists, mechanical engineers, environmental scientists, etc. Ideally, *GreenAI-6G-VEmoBAR*, *GreenAI-6G-VEmoFLOWMAP*, and *GreenAI-6G-EPreS* should be environmentally friendly in practice. When medical items are delivered by UAVs, mobile robots, autonomous vehicles or devices, the amount of carbon emission released by AI execution models needs to be minimized. Furthermore, because electricity usage is linked to carbon emission, the issue of how to lessen the electricity usage expended by UAVs, mobile robots, and autonomous vehicles should be handled continuously. In addition, minimization of electricity usage relies on local electricity infrastructure comprising *Road Side Unit* (*RSU*) that is able to provide replenishment of electricity during stay [13]. Furthermore, after installation of *RSU*, 6G communications among system components can be accelerated as well as be maintained efficiently with a combination of those local infrastructures and components. Hence, the physical and practical installations of local electricity infrastructure and linking between *RSU* and *IAGS* within integrated system are requested toward environmentally friendly 6G convergent system activation.

### 5.3. Open Research Issue 3 of 6G Convergent Green AI

Thirdly, the study is to improve privacy and security toward successful 6G convergent Green AI with secure *GreenAI-6G-VEmoBAR*, *GreenAI-6G-VEmoFLOWMAP*, and *GreenAI-6G-EPreS*. Essentially, the convergent system should be protected against illegal access, serious attacks, information forgery, and compromised system elements. When the 6G convergent Green AI system including *GreenAI-6G-VEmoBAR*, *GreenAI-6G-VEmoFLOWMAP*, and *GreenAI-6G-EPreS* detect a large amount of anonymous information through system components and utilize this information to services, the forgery can present a critical threat. Therefore, the research issue of privacy and security can be considered toward secure realization of 6G convergent AI Green AI system.

## 6. Conclusions

The advent of 6G presents numerous important research directions concerning virtual emotion and epidemic prevention with *Red AI* perspective. Furthermore, the incorporation of *Green AI* perspective into these research directions is indispensable. In this paper, we proposed 6G convergent terrestrial and non-terrestrial infrastructure with two differential views: *Red AI* and *Green AI*. For the *Red-AI*-featured sub-system 1, we defined three favorable applications: *RedAI-6G-VEmoBAR*, *RedAI-6G-VEmoFLOWMAP*, and *RedAI-6G-EPreS*. Furthermore, we highlighted its objectives and operations focusing on accuracy improvement, process speed, and minimum delay. On the other hand, for *Green AI* envisioned sub-system 2, we defined three outstanding applications: *GreenAI-6G-VEmoBAR*, *GreenAI-6G-VEmoFLOWMAP*, and *GreenAI-6G-EPreS*. For each application, we stated the goals and operations with a consideration of task accomplishment, computational expense efficiency, environmental protection, and reduction in spent resources. Furthermore, we provided research roadmaps, open research issues, and directions toward new frontiers in the 6G convergence of virtual emotion and epidemic prevention.

## Data Availability

Data sharing not applicable.

## Abbreviations

The following abbreviations are used in this manuscript:

UAVs	Unmanned Aerial Vehicles
IoT	Internet of Things
